# Function and Therapeutic Potential of Mesenchymal Stem Cells in Atherosclerosis

**DOI:** 10.3389/fcvm.2017.00032

**Published:** 2017-05-22

**Authors:** Feifei Li, Xia Guo, Shi-You Chen

**Affiliations:** ^1^Department of Physiology & Pharmacology, University of Georgia, Athens, GA, USA; ^2^The Department of Cardiovascular Surgery, Union Hospital, Wuhan, China

**Keywords:** atherosclerosis, mesenchymal stem cells, characteristics, functional properties, therapeutics

## Abstract

Atherosclerosis is a complicated disorder and largely attributable to dyslipidaemia and chronic inflammation. Despite therapeutic advances over past decades, atherosclerosis remains the leading cause of mortality worldwide. Due to their capability of immunomodulation and tissue regeneration, mesenchymal stem cells (MSCs) have evolved as an attractive therapeutic agent in various diseases including atherosclerosis. Accumulating evidences support the protective role of MSCs in all stages of atherosclerosis. In this review, we highlight the current understanding of MSCs including their characteristics such as molecular markers, tissue distribution, migratory property, immune-modulatory competence, etc. We also summarize MSC functions in animal models of atherosclerosis. MSC transplantation is able to modulate cytokine and chemokine secretion, reduce endothelial dysfunction, promote regulatory T cell function, decrease dyslipidemia, and stabilize vulnerable plaques during atherosclerosis development. In addition, MSCs may migrate to lesions where they develop into functional cells during atherosclerosis formation. Finally, the perspectives of MSCs in clinical atherosclerosis therapy are discussed.

## Introduction

Atherosclerosis, a vascular disorder leading to occlusion of the arterial wall, causes several important complications including coronary and cerebral artery diseases (1, 2). Smoking, hypertension, diabetes, and dyslipidemia are well-known risk factors for atherosclerosis (3, 4). Despite the effective treatment targeting serum lipid abnormalities, atherosclerosis remains a leading cause of mortality (1, 5). The pathophysiological mechanisms of atherosclerosis are very complex and involve accumulation of lipoprotein aggregates in the subendothelial space, inflammatory responses of vascular endothelial cells (ECs), adhesion and infiltration of monocytes, and transformation of macrophages into foam cells induced by oxidized low-density lipoprotein (ox-LDL) (6–9). In fact, atherosclerosis is a chronic inflammatory disease involving both innate and adaptive immunity. Pattern recognition receptors of innate immunity regulate cholesterol uptake and contribute to the foam cell formation and EC dysfunction (10). Antigen-specific T cells recognizing LDL in the intima are the adaptive immunity components in the development of atherosclerosis and provoke pro-inflammatory stimuli that further exacerbate and propagate this disease (11). Therefore, strategies to mobilize and stimulate immunosuppression may provide novel therapeutic approaches to reduce atherosclerotic cardiovascular disease (12, 13).

Cell therapy has been a focus for intense research and already being used widely for the treatment of various diseases. Mesenchymal stem cells (MSCs), also referred to as multipotent stromal cells, are a class of multipotent stem cells that can be isolated from various tissues including bone marrow, peripheral blood, adipose, placenta, etc. (14–16). MSCs have been explored as an attractive therapeutic agent in various diseases and injury models including acute lung injury, myocardial infarction, acute renal failure, cerebral ischemia, Alzheimer’s disease, and corneal damage (14, 17–22), due to their capabilities of differentiation into multiple cell lineages such as mesodermal lineage (adipocyte, osteoblast, chondrocyte) and myogenic lineage (15, 23). The interests in MSCs have been further enhanced by their advantages over other stem cell populations including their low immunogenicity, easy cultivation, and expansion *in vitro*. Moreover, recent studies reveal the anti-inflammatory properties of MSCs as guardians of inflammation (24). In view of the critical role of inflammatory processes in the initiation and progression of atherosclerosis, adoptive transfer of MSCs, which has the capability to modulate and reduce inflammation, may be a novel therapeutic approach to treat atherosclerosis. Understanding of the activities and functions of MSCs in different atherosclerotic animal models and mechanisms underlying their therapeutic effects may be important for further investigation into its potential clinical application.

## Characteristics of MSCs

### Tissue Sources, Isolation, and Expansion of MSCs

Mesenchymal stem cells have been found in almost all tissues/organs and can be derived from a variety of different sources, including adult peripheral blood, adipose tissue, and bone marrow, as well as fetal tissues, e.g., umbilical cord blood, Wharton’s jelly, amnion, amniotic fluid, and placenta (16). Currently, most MSCs used for clinical trials are isolated from bone marrow, adipose tissue, and umbilical cord blood (25). Bone marrow-derived mesenchymal stem cells (BM-MSCs) first described by Friedenstein et al. are the most frequently investigated cell type and often considered as the gold standard (26). However, the procedure for bone marrow aspirate is highly invasive for patients and accompanied by a risk of infection. Moreover, the limited accessibility is coupled with a relatively low cell yield (0.001–0.01%) and a substantial reduction in the expansibility of the cells in aged populations (27). Therefore, alternative sources of MSCs have been studied for their biological properties, differentiation capacities, and surface marker expression. The second major source of MSCs is adipose tissue-derived stem cells (AT-MSCs). AT-MSCs are normally isolated from biological materials generated during liposuction, lipoplasty, or lipectomy procedures. These cells share many biological characteristics with BM-MSCs (28). Although the colony frequency of cells obtained from adipose tissues is higher than those of bone marrow, there is controversy on whether AT-MSCs are truly MSCs, as they are often named as “adipose tissue stem cells” (29, 30). Embryonic tissues are also important sources for MSC isolation. MSCs from the embryonic tissues have superior biological properties compared with BM-MSCs (16). They also have improved proliferative capacity, life span, and differentiation potential compared with MSCs derived from adult tissues (16).

Several different procedures have been used to isolate MSCs from different tissues. The commonly applied methods for preparing BM-MSCs or MSCs derived from umbilical cord blood (UBC-MSCs) utilize density gradient isolation or direct plating to separate mesenchymal from hematopoietic cells by their adhesion capacities to the plastic surface (31). Seeding densities are very important for successfully expanding MSCs. Accordingly, plating densities of 4–22 × 10^3^ bone marrow mononuclear cells/cm^2^ can yield up to 9.8 × 10^8^ MSCs when they are harvested after one passage (32). The UBC-MSCs fraction is suggested to be seeded at a higher density of 1 × 10^6^/cm^2^ due to their low frequency (29). To isolate MSCs from adipose tissue, enzymatic treatment is commonly used. Centrifugation is performed to acquire the preadipocyte stromal vascular fraction, and the adipocyte fraction is removed. After culture for 10 days, approximately 1:1,000 cells within the stromal vascular fraction will generate colony-forming units (29).

### Validation of MSC Identity

Mesenchymal stem cells are identified by a combination of phenotypic and functional characteristics. In line with the International Society for Cellular Therapy, MSCs must be plastic adherent under basic culture conditions and able to differentiate to adipocytes, chondrocytes, and osteoblasts *in vitro*. Numerous publications have suggested minimal criteria of several stem cell markers to categorize MSCs, including the positive expression of CD29, CD44, CD73, CD90, and CD105 and the negative expression of hematopoietic markers (CD14, CD34, CD45), endothelial markers (CD31), human leukocyte antigen (HLA) class II, costimulatory molecules (CD80, CD86), and HLA-DR surface molecules (33–35). These biomarkers constitute a uniform characterization of MSCs and enable the comparison of different studies. However, some of these markers may be lost, or new markers may arise during culture process. The markers may also vary between different sources. For example, fibroblast-derived MSCs express CD34 and CD45 surface markers, which are absent in BM-MSCs (36). UBC-MSCs showed CD45, CD14, and CD31 positive and CD34, CD1a, and CD80 negative, which are quite different from that of the BM-MSCs (37–39).

### Distribution and Migration of MSCs

Mesenchymal stem cells are increasingly used as an intravenously administered cellular therapy due to their characteristics of migration to the site of injury. To track the distribution of MSCs *in vivo*, various methods of labeling have been used, including radioactive labeling, fluorescent vital dyes, contrast agents, transduction with reporter genes, and the use of donor cell-specific DNA markers such as microsatellites (40–44). By using these approaches, MSCs are found to migrate to a variety of tissues after the intravenous injection although low or very low frequencies of MSCs are detectable in these tissues (45). Early after administration of the MSCs, signals from the injected cells are found at the highest frequencies in the lung followed by liver and spleen (46, 47). Due to the limitation of technologies detecting transplanted cells, there is only limited evidence indicating that the MSCs migrate as intact cells into their target locations. Intravenously injected MSCs have been observed to accumulate in tissues of myocardial ischemia, and adhesion molecules very late antigen-4 and vascular cell adhesion molecule-1 appear to be involved in the migration (48–50). Moreover, an increased level of CC-chemokine ligand (CCL)-2 has been shown to facilitate the accumulation of MSCs in heart (51). In murine stroke model, MSCs migrate into ischemic areas after intravenous delivery, which involves the endothelial expression of P- and E-selectin (52). Apparently, the route of administration is critical for the efficiency of MSC therapy. Compared with the intravenous route, intra-arterial route of administration is more effective in avoiding pulmonary entrapment of MSCs, and may thus improve the biodistribution and bioavailability of transplanted MSCs to clinically relevant tissues (53, 54).

### Immunomodulatory Properties of MSCs

A number of studies have confirmed the immunosuppressive function of MSCs. MSCs have been used to treat severe graft-versus-host disease based on the fact that MSCs can alter several properties of T cells. The most prominent one is to efficiently suppress the proliferation of the activated CD4^+^ T helper cells and CD8^+^ cytotoxic T cells (55, 56). Indoleamine-pyrrole-2-3-dioxygenase (IDO), an intracellular enzyme, is the primary mediator of MSC immunomodulatory activity. IDO has been shown to reduce immune cell proliferation by regulating tryptophan depletion and accumulating metabolites such as kynurenine (57, 58). MSCs are also known to halt B-cell maturation in G0/G1 phase and simultaneously diminish their chemotactic activity. In addition, MSCs can block the maturation of dendritic cells (DCs), resulting in a reduced expression of antigens and costimulatory molecules necessary for activating T-cells (59). Moreover, MSCs are found to downregulate the activating receptors of natural killer (NK) cells NKp30, NKp44, and NKG2D (60).

Several studies suggest that MSCs alter the cytokine secretion profile of immune cells including DCs, naive and effector T cells, and NK cells (61). Indeed, MSCs can reduce tumor necrosis factor α (TNF-α) secretion from mature DCs type 1 (DC1), increase interleukin 10 (IL-10) secretion from mature DC2, decrease Interferon gamma (IFN-γ) secretion from NK cells and Th1 cells, and increase IL-4 from Th2 cells. In addition, MSCs inhibit the production of IL-17, IL-22, IFN-γ, and TNF-α by preventing the differentiation of naive CD4^+^ T cells into Th17 cells *in vitro* (62). Moreover, BM-MSCs decrease the production of pro-inflammatory cytokines IFN-γ, TNF-α, and IL-2 in T and B lymphocytes and suppress cell proliferation (63). MSCs have also been shown to secrete tumor necrosis factor alpha-stimulated gene-6 (TSG-6), a powerful anti-inflammatory factor (18). Toll-like receptors (TLRs) such as TLR3 and TLR4 are abundantly expressed in MSCs, and their activation regulates MSC anti-inflammatory functions (64).

## Function of MSCs in Atherosclerosis

### The Pathophysiological Mechanisms of Atherosclerosis

Atherosclerosis has traditionally been considered as a metabolic disorder caused by hyperlipidemia and fatty deposits and a chronic inflammatory disease of the arterial wall (12). Inflammation plays a crucial role in every stage of atherosclerosis from initial onset of the plaque to rupture. Early in the disease process, entrapped ox-LDL in the vessel wall leads to arterial endothelial dysfunction and an upregulation of leukocyte adhesion molecules such as selectins, integrins, and immunoglobulin proteins, which induce inflammatory cell adhesion, rolling, and migration to subendothelial region (65–67). Thereafter, monocytes, T cells, and neutrophils infiltrate through gaps between interendothelial junctions. The monocyte-derived macrophages and DCs engulf lipid molecules and then become foam cells, and simultaneously produce an array of inflammatory cytokines (68). Accumulation of immune cells and lipid droplets in the intima result in the early plaque, known as fatty steak. In the center of a mature plaque, foam cells and extracellular lipid droplets form a core region surrounded by a cap of smooth muscle cells (SMCs) and a collagen-rich matrix (7). Both macrophages and DCs express TLRs to mediate the activation of antigen-presenting cells and production of inflammatory cytokines. CD4^+^ T cells are crucially involved in the development of atherosclerosis, and their depletion reduces the lesion size by 70% (69). The predominant T cell subset in human and murine atherosclerotic lesions is the Th1 subset, which produces a number of inflammatory cytokines such as IFN-γ (70, 71). IFN-γ promotes vascular inflammation by enhancing maturation and activation of antigen-presenting cells, increasing macrophage lipid uptake, reducing collagen production by phenotypically modulated SMCs, and enhancing expression of endothelial adhesion molecules to facilitate leukocyte recruitment to the lesions (72). The continuous recruitment of leukocytes to atherosclerotic arteries leads to a feed-forward promotion of inflammatory cycle.

Due to the essential role of inflammation in the initiation and progression of atherosclerosis, MSCs transplantation, which has the capacity to modulate and reduce inflammation, has been broadly explored as a therapeutic approach to treat atherosclerosis. The notable characteristics of allogeneic MSCs, such as low immunogenicity, inhibition of T cell proliferation, and memory T cell responses, make allogeneic MSCs transplantation an attractive approach (56, 73, 74). Multiple studies have demonstrated that MSCs exhibit atheroprotective effects in animal atherosclerosis, mostly induced by high-fat diet in apolipoprotein E (ApoE) or low-density lipoprotein receptor (LDLR) knockout mice. In most of these studies, MSCs are derived from bone marrow although umbilical cord blood-derived MSCs and skin-derived MSCs (S-MSCs) are also used (Table 1).

**Table 1:** **Mesenchymal stem cell (MSC) treatments against atherosclerosis in animal models**.

Reference	MSCs sources/dose	Route	Animal model	Mechanism of MSC action
Fang et al. (75)	Bone marrow	Allogeneic	New Zealand rabbits	PAI-1, hs-CRP, MMPs ↓
	1 × 10^7^ cells	Intra-arterial		Collagen fibers ↑

Wang et al. (76)	Bone marrow	Allogeneic	ApoE^−/−^ mice	CD4^+^CD25^+^FOXP3^+^ Tregs ↑
	10^7^ cells	Intravenous		CD36, SRA ↓

Lin et al. (77)	Bone marrow	Allogeneic	ApoE^−/−^ mice	IL8, MIP-2, eNOS ↑
	2 × 10^5^ cells	Intravenous		

Frodermann et al. (78)	Bone marrow	Allogeneic	LDLR^−/−^ mice	Circulating monocytes ↓
	0.5 × 10^6^ cells	Intravenous		CD4^+^ T cells, CCL2, IFN-γ↓
				TNF-α, serum cholesterol level ↓
				Tregs ↑

Wang et al. (79)	Bone marrow	Allogeneic	New Zealand rabbits	hs-CRP, TNF-α, IL-6, NF-κB ↓
	1 × 10^7^ cells	Intravenous		MMPs, cell apoptosis ↓
				TSG-6, IL-10 ↑

Abdel-Kawi and Hashem (80)	Cord blood	Allogeneic	Albino rats	iNOS ↑
	3 × 10^6^ cells	Intravenous		

Li et al. (81)	Skin	Allogeneic	ApoE^−/−^ mice	PGE2, IL-10 ↑
		Intravenous		TNF-α, NF-κB ↓

### MSCs Modulate Cytokine and Chemokine Secretion during Atherosclerosis Development

The protective effects of MSCs in animal atherosclerosis models are mainly attributable to its production of a number of anti-inflammatory factors. The application of BM-MSCs in atherosclerotic mouse causes an increased secretion of anti-inflammatory cytokines such as transforming growth factor (TGF)-β1 and IL-10, and the decreased production of pro-inflammatory cytokines, such as TNF-α, IL-1β, and IL-6 (76). TGF-β1 is involved in the MSC-mediated induction of CD4^+^CD25^+^Foxp3^+^ regulatory T cells (Tregs) (82) and the decreased proliferation of NK cells (83). IL-10 exerts its anti-atherogenic effects primarily by influencing the local inflammatory process within the lesion through inhibiting macrophage activation, matrix metalloproteinase, and pro-inflammatory cytokines (65, 84). Frodermann et al. have found that MSC therapy significantly reduces serum CCL2 levels, a chemokine that attracts and activates mononuclear cells (78). In addition, MSC treatment results in an overall reduced inflammatory state as well as a significant reduced differentiation of naive T cells. Similarly, treatment with skin-derived MSCs (S-MSCs) reduces the release of TNF-α and increases the expression of IL-10 both *in vivo* and *in vitro*, which is both dependent on NF-κB activation (81). There is a reduced expression of NF-κB in atherosclerotic plaque after MSC transplantation (79). These findings are consistent with many other studies showing that MSCs can inhibit the expression and activity of NF-κB (85–87).

Mesenchymal stem cell activation also leads to the production and release of modulation of target molecules including IDO, prostaglandin E2 (PGE2), and TSG-6 (88). IDO inhibits Th17 differentiation through an exhaustion of tryptophan (58). Moreover, IDO decreases the proliferation and cytotoxic activity of NK cells activated by IL-2 in the presence of MSCs and inhibits the maturation and functional activity of DCs (89). PGE2 has been shown to diminish T cell proliferation, stimulate IL-4 and IL-10 secretion, and promote CD4^+^CD25^+^Foxp3^+^ Tregs differentiation (90). TSG-6 is not constitutively expressed in normal tissues or cells, but is upregulated in response to pro-inflammatory cytokines such as TNF-α, IL-1, and IL-6 (91, 92). TSG-6 facilitates a feedback mechanism to inhibit inflammation-mediated extracellular matrix remodeling by reducing inflammatory factor expression and inhibiting neutrophil infiltration and plasmin activity (93, 94).

### MSCs Improve Endothelial Function during the Development of Atherosclerosis

Endothelial dysfunction is one of the earliest events of atherosclerosis, resulting in subsequent lipid accumulation, macrophage recruitment, foam cells formation, and T cell and platelet recruitment (95). In addition to providing a lining for vessel walls, the endothelium is a complex endocrine and paracrine organ that plays a crucial role in the maintenance of vascular homeostasis. Endothelial nitric oxide synthase (eNOS) is responsible for the production of most vascular nitric oxide (NO) (96, 97). NO acts as a local vasodilator by increasing smooth muscle cyclic guanosine monophosphate (cAMP) levels while inhibiting leukocyte adhesion and activation, platelet aggregation, and SMC proliferation. NO also has anti-inflammatory properties by inhibiting the expression of leukocyte adhesion molecules (98). Although ECs have the potential to self-repair in response to inflammatory stimuli, MSCs appear to be able to accelerate the repairing process. For example, amnion-derived MSCs have been reported to enhance EC viability as shown by decreasing lactate dehydrogenase level and stabilize the endothelial network formation *in vitro* (99). Lin et al. demonstrated that allogeneic BM-MSCs transplantation attenuates atherosclerosis through repairing the diseased endothelium and improving endothelial function (77). ox-LDL deactivates Akt/eNOS activity, induces eNOS degradation, and thus inhibits NO production in EC. However, coculture with human MSCs reverses the effects of ox-LDL on ECs. It appears that the protective effect of MSCs on EC activation of the Akt/eNOS pathway is achieved mainly through upregulation of IL8 and macrophage inflammatory protein (MIP)-2. The effects of human/mouse MSCs on ox-LDL-treated ECs are blocked by the neutralization antibodies against IL8/MIP-2. Therefore, MSC transplantation could ameliorate atherosclerosis by improving endothelial function and plaque formation.

### MSCs Increase the Quantity and Enhance the Function of Tregs during Atherosclerosis Development

Regulatory T cells have been shown to exert an immunosuppressive function through producing inhibitory cytokines such as IL-10 and TGF-β. Tregs mediate cell–cell contact by membrane-bound TGF-β and cytotoxic T lymphocyte-associated antigen (100, 101). Tregs are initially characterized as CD4^+^CD25^+^ T cells. However, later studies identify forkhead box transcription factor (FOXP3) as a key lineage protein and a master regulator in Treg development and function (102–104). In atherosclerotic plaques, there is a low number of FOXP3^+^ Tregs (105, 106). Knockdown of FOXP3 promotes the progression of atherosclerosis in mice (106, 107), suggesting a possible atheroprotective function of FOXP3^+^ Tregs. Transplantation experiments in immune-deficient, hyperlipidemic mice demonstrate that Tregs exert their atheroprotective role by repressing the function of DCs and Th1/Th2 cells (108). Mechanistic analyses reveal that IL-10 and TGF-β, the two major cytokines produced by Tregs, suppress functions of DCs and Th1/Th2 cells in atherosclerosis (109–112). In addition, Tregs are able to repress the expression of the matrix metalloproteinases MMP-2 and MMP-9, two important proteinases degrading extracellular matrix proteins, and thus enhance the lesion stability in atherosclerosis mouse model (107, 108, 113).

The major obstacle associated with Treg treatment is the inability to efficiently isolate a pure population of Tregs. There are no validated cell surface markers that can be used for cell sorting. A promising alternative is to use MSCs. MSCs can recruit and promote the generation of Tregs (75, 88). Indeed, MSC treatment has significantly increased the number and function of Tregs in cultured splenocytes. It also increases the mRNA and protein expression of FOXP3 in atherosclerotic mouse model (76). It is also reported that ApoE^−/−^ mice treated with multiple times and high numbers (10^7^) of MSCs can dramatically reduce the long-term overall loading of effector T cells, which is partially due to the lasting increase of Tregs. Several mechanisms underlie the MSC-induced expansion of Tregs. Soluble factors are proved to be involved while cell–cell contact is also vital. MSCs are able to induce Tregs by directly contacting with CD4^+^ T cells (82). Melief et al. have also reported that MSCs increase TGF-β1 secretion to promote the generation of Tregs (114). Further study reveals that the increased gene expression of the Notch ligand, Delta-like 1, is essential for the augmented Tregs induction by TLR-activated MSCs, which is dependent on cell–cell contact (64). Notch ligand Jagged-1 is also involved in MSC induction of Tregs (115). In addition, the presence of monocytes is important for the MSC-induced generation of Tregs as well. MSCs promote monocyte survival and induce monocytes to differentiate toward an anti-inflammatory type 2 macrophage phenotype, which mediates Tregs formation by the production of CCL18 (114).

### MSCs Can Migrate to Lesions and Develop into Functional Cells during Atherosclerosis Development

The intrinsic ability of MSCs to differentiate into functional cell types enables them to repair the diseased or injured tissues in which they are localized. The carboxyfluorescein succinimidyl ester-labeled mMSCs are found in areas close to the endothelium at 7 days after the injection while S-MSCs migrate to atherosclerotic plaque and selectively take up residence near macrophages (77, 81). The transplanted BM-MSCs are able to “home in” on the ruptured plaque regions and differentiate into ECs and collagen fibers (75). BrdU-labeled BM-MSCs are observed in ruptured plaques 4 weeks after the MSC transplantation. The mechanisms regulating stem cell differentiation and their mobilization or migration to the site of vascular injury involve several mediators and receptors, such as P-selectin glycoprotein ligand-1, α4 integrin, CXC chemokine receptor-2 and -4, and β1- and β2-integrins (116, 117). In addition, the interaction of platelets with progenitor cells also influences MSC chemotaxis, adhesion, activation, and differentiation into mature ECs during vascular repair (118).

### MSCs Reduce Dyslipidemia during the Development of Atherosclerosis

Dyslipidemia is a major risk factor for the development and progression of atherosclerosis (119). MSCs appear to have indirect effects on cholesterol metabolism through immune modulation. The connection between immune cells and cholesterol metabolism has been established. Both ApoE^−/−^ mice on chow diet and LDLR^−/−^ mice on high-fat diet lacking both B and T cells show reduced plasma lipoproteins, especially the apoB-containing lipoproteins (120). MSCs treatment not only suppresses inflammatory responses but also significantly lowers the plasma cholesterol levels in MSC-treated mice due to a reduction of VLDL levels after a 5-week treatment (78). In MSC-treated mice, a significant decrease of lipoprotein lipase is observed in liver, which reduces the availability of free fatty acids for VLDL synthesis. MSC-treated mice also exhibit a reduced VLDL metabolism due to the reduced activation of Kupffer cells. Kupffer cells can express mediators promoting VLDL secretion by hepatocytes (121). Moreover, lipoprotein lipase deficiency in macrophages reduces their uptake of VLDL or ox-LDL, thereby attenuating atherosclerosis (122, 123). Overall, MSCs reduce VLDL levels by decreasing inflammation. Indeed, TNF-α that is downregulated in MSC and splenocyte coculture has been shown to upregulate SREBP-1c, which increases VLDL synthesis (124). Conversely, IL-10 overexpression reduces plasma cholesterol, mostly due to a reduced VLDL, in LDLR^−/−^ mice (125).

### MSCs Enhance Stability of Advanced Atheroslerotic Lesions

Atherosclerotic plaques are able to alternate between stable and vulnerable states depending on the internal environment. It is generally believed that lesions with a thin fibrous cap, large lipid core, and a large number of macrophages are unstable and prone to rupturing. Rupture and embolism of the atherosclerotic plaque lead to acute coronary syndrome and ischemic stroke (126, 127). Clinical evidence indicates that the instability rather than the plaque size determines the prognosis of cardiovascular diseases (128, 129). Recently, allogeneic MSCs have been evaluated for their potential to repair ruptured lesions. It appears that MSCs can increase the regeneration of the inner endothelial lining and collagen fiber formation in the vessel wall, implying their potential in treating advanced lesions (75).

C-reactive protein (CRP) is recognized as a predictive indicator of plaque instability because of its direct pro-inflammatory effects. CRP induces ECs to express various adhesion molecules and chemotactic molecules. CRP also stimulates monocytes to produce and secrete potent pro-coagulant factors promoting the inflammatory response (130, 131). Plasminogen activator inhibitor-1 (PAI-1) is a major inhibitor for fibrinolysis and an important risk factor for thrombotic diseases (132). In a rabbit advanced atherosclerosis model, MSCs transplantation dramatically decreases the expression of sensitive biomarkers for tissue damage, including CRP, MMPs, and PAI-1 (75). TNF-α promotes inflammatory cell accumulation in atherosclerotic plaques to negatively impact plaque stability and promote thrombosis and cell necrosis (133). IL-10, on the other hand, promotes lesion stability through inhibiting inflammatory cell accumulation and inducing SMC proliferation (109, 134, 135). MSC transplantation effectively stabilizes vulnerable plaques in atherosclerotic rabbit model through immune modulation, as indicated by a reduction in TNF-α and IL-6 and an increase in IL-10 in MSC-treated animals (79). Moreover, the expression of MMP-1, MMP-2, and MMP-9 in lesion is decreased after MSC transplantation, suggesting that alterations in MMPs may influence the extracellular matrix and further affect the lesion stability. These findings indicate that MSCs may alter plaque vulnerability by decreasing the regional collagen degradation via reduction of MMP synthesis. Furthermore, the cell apoptosis is one of the major features in atherosclerotic plaque (136, 137). In fact, the apoptosis of vascular ECs, vascular SMCs, and macrophages is involved in the formation, development, and rupture of atherosclerotic plaque (138). MSC transplantation dramatically decreases the number of apoptotic cells in plaques, suggesting that MSCs may increase the plaque stability also by inhibiting cell apoptosis (79).

## Pitfalls in Using MSCs to Treat Atherosclerosis

Numerous clinical and preclinical trials have suggested that MSC transplantation is safe (139–141). The minimal and maximal dosages for therapeutic application has not been determined yet, but currently applied doses are in the range of <1–5 × 10^6^ MSCs/kg body weight (142). Koç et al. have found that intravenously adoptive MSCs are well-tolerated in patients with mammary carcinoma at a dose of 1 × 10^6^ MSCs/kg body weight (143). However, in animal models, allogeneic MSCs can be rejected by recipient mice although allogeneic MSCs show low immunogenicity (144). In addition, although preclinical and early phase clinical trials have not detected potential pitfalls of MSC therapy in human patients after MSC transplantation, tumor formation has been reported in several rodent models. BM-MSC transplantation may lead to the occurrence of gastric cancers (145). The chromosomal instability is also observed in mouse BM-MSCs that may lead to a malignant transformation (146). In October 2011, the experts in Cell Products Working Party have reached a consensus that good manufacturing practice conditions, such as cell preparation, culture, and manipulation, decrease the risk of tumorigenicity in MSC therapy. Cell culture condition and cell propagation duration significantly impact the formation of cytogenetic abnormalities (147).

## Conclusion and Perspective

The major advantage of MSCs is their ability to migrate to sites of injury, respond dynamically to the extent of injury, and secrete a broad range of beneficial factors. These factors can modulate the inflammation status and restore endothelium function in atherosclerotic lesions. MSC transplantation represents a novel approach for efficient prevention and treatment of atherosclerotic plaque rupture. Studies on MSCs have provided theoretical and experimental evidence for its clinical application. However, there are no differences in atherosclerotic plaque burden after long-term observations in the MSC treatment group compared to controls, which indicates that the therapeutic effects of MSCs may not sustain for a long time after the removal of engrafted MSCs. To acquire persistent effects of MSCs, multiple treatments may be necessary. In addition, to effectively translate the current findings into clinic, future studies using mouse models with humanized immune system may be necessary, such as severe combined immunodeficiency (SCID), NOD/Shi-scid, IL-2Rγ null (NOG), or NOD/Lt-scid, IL-2Rγ null (NSG) mice.

Overall, much investigation remains to be done before understanding how MSCs are distributed and removed in the recipient, and whether their atheroprotecive effect is mediated through immunomodulation, or if the effectiveness of MSC therapy can be enhanced by various pretreatments with cocktails of different factors.

## Author Contributions

FL and XG wrote the review and S-YC revised the review.

## Conflict of Interest Statement

The authors declare that the research was conducted in the absence of any commercial or financial relationships that could be construed as a potential conflict of interest.
